# Multispecies Evaluation of a Long-Acting Tenofovir Alafenamide Subdermal Implant for HIV Prophylaxis

**DOI:** 10.3389/fphar.2020.569373

**Published:** 2020-11-25

**Authors:** Manjula Gunawardana, Mariana Remedios-Chan, Debbie Sanchez, Simon Webster, Patricia Galvan, Rob Fanter, Amalia E. Castonguay, Paul Webster, John A. Moss, Joseph Kuo, Philippe A. Gallay, Kathleen L. Vincent, Massoud Motamedi, Dana Weinberger, Mark A. Marzinke, Craig W. Hendrix, Marc M. Baum

**Affiliations:** ^1^ Department of Chemistry, Oak Crest Institute of Science, Monrovia, CA, United States; ^2^ Department of Immunology and Microbiology, The Scripps Research Institute, La Jolla, CA, United States; ^3^ Center for Biomedical Engineering, University of Texas Medical Branch at Galveston, Galveston, TX, United States; ^4^ Approva Consulting LLC, Boulder, CO, United States; ^5^ Department of Medicine, Johns Hopkins University, Baltimore, MD, United States; ^6^ Department of Pathology, Johns Hopkins University, Baltimore, MD, United States

**Keywords:** pre-exposure prophylaxis, long-acting, sustained release, tenofovir alafenamide, subdermal implant, HIV prevention

## Abstract

New HIV-1 infection rates far outpace the targets set by global health organizations, despite important progress in curbing the progression of the epidemic. Long-acting (LA) formulations delivering antiretroviral (ARV) agents for HIV-1 pre-exposure prophylaxis (PrEP) hold significant promise, potentially facilitating adherence due to reduced dosing frequency compared to oral regimens. We have developed a subdermal implant delivering the potent ARV drug tenofovir alafenamide that could provide protection from HIV-1 infection for 6 months, or longer. Implants from the same lot were investigated in mice and sheep for local safety and pharmacokinetics (PKs). Ours is the first report using these animal models to evaluate subdermal implants for HIV-1 PrEP. The devices appeared safe, and the plasma PKs as well as the drug and metabolite concentrations in dermal tissue adjacent to the implants were studied and contrasted in two models spanning the extremes of the body weight spectrum. Drug and drug metabolite concentrations in dermal tissue are key in assessing local exposure and any toxicity related to the active agent. Based on our analysis, both animal models were shown to hold significant promise in LA product development.

## Introduction

Long-acting (LA) biomedical devices delivering antiretroviral (ARV) drugs locally or systemically reduce dosing frequency and, consequently, may lead to increased product adherence (Krogstad et al., 2019) and effectiveness. The strategy is being exploited in HIV-1 pre-exposure prophylaxis (PrEP) using injectable formulations and subdermal implants delivering ARV agents for four weeks, or longer (Lykins et al., 2017). The prodrug tenofovir alafenamide (TAF) has the potency required to make a subdermal implant theoretically feasible (Gunawardana et al., 2015), and the clinical pharmacology of the parent drug tenofovir (TFV) and its active metabolite against HIV-1, TFV diphosphate (TFV-DP) are well understood. It is therefore not surprising that we (Gunawardana et al., 2015) and others (Schlesinger et al., 2016; Chua et al., 2018; Johnson et al., 2019; Su et al., 2019) are developing a range of complementary subcutaneous TAF implant technologies.

The choice of animal model and realistic human scaling of pharmacokinetic (PK) and pharmacodynamic (PD) measures are critical for successful preclinical development of LA drug delivery products for HIV-1 PrEP. Here, we compared the PKs of TAF delivery from a subdermal implant in two animal models at opposite ends of the body weight spectrum: mice and sheep. This is the first report of the application of these models to the evaluation of TAF implants.

## Materials and Methods

### Materials and Chemicals

TAF, as the free-base, was kindly provided by Gilead Sciences, Inc. (Foster City, CA). Medical-grade silicone tubing was custom-manufactured by Trelleborg Healthcare and Medical (Los Robles, CA). All other chemicals and reagents were purchased as described previously (Gunawardana et al., 2015), unless otherwise noted.

### Tenofovir Alafenamide Implant Fabrication

Mouse-sized (length, 10 mm) TAF implants were fabricated using methods described previously (Gunawardana et al., 2015). In the current study, TAF was compacted into microtablets without excipients using a pellet press (Globe Pharma MTCM-I, North Brunswick, NJ), as described in the literature (Kuo and Kuzma, 2010; Gunawardana et al., 2014). Each implant contained on average 24 mg TAF. *In vitro* release studies using single implants were carried out as described previously (Gunawardana et al., 2015).

### Animal Studies

Animal studies were carried out at The Scripps Research Institute (C57BL/6J mice) and University of Texas Medical Branch at Galveston (merino sheep). Animals were handled in strict accordance with the Guide for the Care and Use of Laboratory Animals (National Research Council, 2001), under approved internal Institutional Animal Care and Use Committee protocols using internal Standard Operating Procedures. The devices were implanted subcutaneously either surgically at the backside of the vertebral column in the dorsal scapular region (mice) or *via* sterile trocar to the lateral neck (sheep).

### Safety Assessment

Toxicity was evaluated by clinical observations, cage-side observations (at least once daily), and body weight (at least weekly). Formaldehyde-fixed dermal tissue specimens (one per animal) collected on study Day 21 (mouse, *vide infra*) and Day 14 (sheep, excisional biopsy) were paraffin-embedded, sectioned, and H&E stained using established methods. The slides were evaluated for microscopic findings by a certified pathologist (Vet Path Services, Inc., Mason, OH). Histopathology grades were assigned as grade 1 (minimal), grade 2 (mild), grade 3 (moderate), grade 4 (marked), or grade 5 (severe) based on an increasing extent of overall change.

### Animal Study Design

Mice (*N* = 3) were sacrificed at each timepoint (0, 7, 14, and 21 d), exsanguinated, and the blood converted to plasma. The implant and surrounding capsule were removed while still encased in a block (ca. 2 × 1 × 1 cm) of associated tissue in accordance with ISO 10993-6 guidelines (ISO, 2016). The capsule was cut longitudinally and rolled open, taking care not to disrupt the architecture of the tissue. The implant was removed for residual drug analysis to determine *in vivo* TAF release rate according to published methods (Gunawardana et al., 2015). A portion of the tissue was placed into 4% paraformaldehyde solution (phosphate-buffered at pH 7.2), and stored at 4°C, for histopathology (*vide supra*) and another was flash-frozen for drug concentration analysis.

Sheep (*N* = 4) were used in a non-terminal study and blood was collected at each timepoint (0, 1, 7, and 14 d) and converted to plasma. Two dermal tissue biopsies adjacent to the implant (within 4 mm) were collected on Day 14 and preserved for histopathology and bioanalysis as described above. Used implants were retrieved for residual drug measurement to determine *in vivo* TAF release rate. In the mouse studies, *in vivo* TAF release rates were obtained by plotting the cumulative mass of TAF released (mg, *y*-axis), calculated from the amount of drug remaining in used implants, *vs.* the time the implant was in place (d, *x*-axis). A simple linear regression analysis of the data afforded the *in vivo* TAF release rate (mg d^−1^) as the slope. In the sheep studies, the mass of drug released was divided by the period of implant use (14 d) to calculate *in vivo* TAF release rate.

*In situ*, non-invasive ultrasonic imaging of the implants during the sheep study was carried out using a Vevo 2100 high-resolution ultrasound system (Fujifilm VisualSonics, Toronto, ON) with a 40 MHz linear array transducer.

### Bioanalysis

Drug concentrations in plasma (TAF, TFV) and dermal tissue (TFV, TFV-DP) samples were measured using LC-MS/MS as described previously (Gunawardana et al., 2015; Hummert et al., 2018). Mouse plasma was analyzed at Oak Crest with lower limits of quantification (LLQ) for TAF and TFV in plasma of 0.5 ng ml^−1^ and 5 ng ml^−1^, respectively. The remaining samples were analyzed by the Clinical Pharmacology Analytical Laboratory at the Johns Hopkins University School of Medicine with the following LLQs: sheep plasma: TAF, 0.03 ng ml^−1^; TFV, 1 ng ml^−1^; dermal tissue: TFV, 0.05 ng/sample; TFV-DP, 5 fmol/sample. Tissue results were normalized to weight and reported as ng mg^−1^ or fmol mg^−1^, respectively, and the median sample weight in the sheep study was 119 mg.

## Results

### *In Vitro* and *in Vivo* Tenofovir Alafenamide Release Rates

**FIGURE 1:**
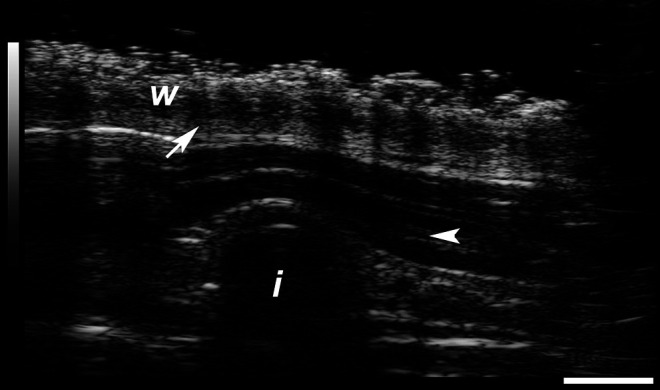
Ultrasound image recorded at 40 MHz showing tenofovir alafenamide implant (end-on view) in subcutaneous layer during sheep study (animal P564, Day 0). *w*, top wool layer; *i*, implant; arrow, epidermis; arrowhead, dermis. Scale bar, 2 mm.

TAF Implants formulated for preclinical evaluation afforded linear drug release *in vitro* (dissolution rate, *K*_*d*_ 1.26 ± 0.12 mg d^−1^ over 21 d). Implants with these characteristics were evaluated in C57BL/6J mice and merino sheep, and the corresponding *in vivo* TAF release rates were 0.23 ± 0.07 mg d^−1^ and 0.30 ± 0.04 mg d^−1^ (mean ± SEM), respectively. Over 97% of the material in the used implants remained as TAF (mice, 21 days; sheep, 14 days). An ultrasound image of an implant in place during the sheep study is shown in Figure 1.

### Safety Assessment

No adverse events related to treatment with the test article were noted during the course of the studies. Dermal tissue specimens collected adjacent to the implant on study Day 21 (mouse) and Day 14 (sheep) were sectioned, H&E stained for microscopic imaging, and analyzed for clinical evaluation by a certified pathologist. The mouse study samples displayed no visible lesions to skeletal muscle, had minimal mononuclear and neutrophilic inflammation associated with fat and fascia, and had a capsule where the implant had been. Three out of the four sheep study samples displayed minimal mononuclear perivascular infiltrates in the skin/subcutis on Day 14 after implantation, with no visible lesions for the fourth sheep. The alteration was not considered adverse.

### Multispecies Pharmacokinetics

**FIGURE 2:**
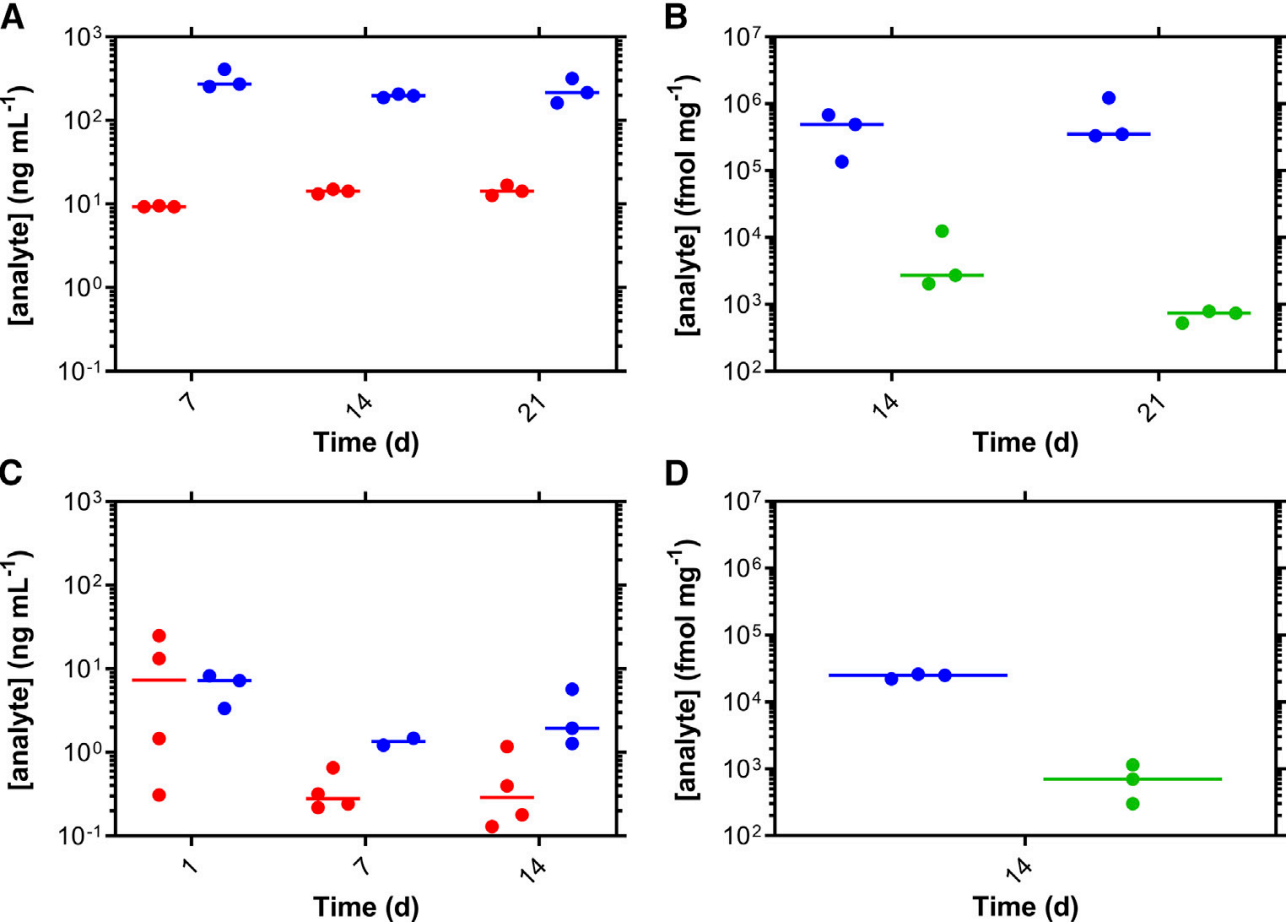
Subdermal implantation of tenofovir alafenamide (TAF) long-acting devices in mice **(A,B)** and sheep **(C,D)** maintains sustained drug levels. Pharmacokinetic profiles of plasma TAF (red circles) and TFV (blue circles) are presented for mice **(A)** and sheep **(C)**. Molar concentrations of TFV (blue circles) and TFV-DP (green circles) in dermal tissues collected adjacent to the implants are shown for mice **(B)** and sheep **(D)**. Each data point represents one animal sample; horizontal lines correspond to the median.

TAF and TFV plasma concentrations as well as TFV and TFV-DP concentrations in dermal tissues collected adjacent to the implant are described in Table 1; Figure 2. It should be noted that TAF is unstable in plasma, especially mouse plasma, and rapidly converts to TFV (Parsons et al., 2020). Median (IQR), paired TFV:TFV-DP molar ratios in dermal tissues for the mouse and sheep studies were 407 (121–591) and 37 (28–60), respectively. Median, paired TFV (dermal tissue):TFV (plasma) ratios for the mouse and sheep studies were 670 and 3,681, respectively.

**TABLE 1:** Summary of TAF, TFV, and TFV-DP concentrations from the mouse (TAF release rate 0.23 ± 0.07 mg d^−1^) and sheep (TAF release rate 0.30 ± 0.04 mg d^−1^) studies at equilibrium.

Animal, analyte, matrix^a^	n	% above LLOQ^b^	Median (IQR^c^)
Mouse, TAF, plasma	9	100	
ng mL^−1^			13.2 (9.6–14.3)
Mouse, TFV, plasma	9	100	
ng mL^−1^			215.7 (198.3–275.2)
Mouse, TFV, dermal tissue	6	100	
ng mg^−1^			122.0 (97.1–182.6)
fmol mg^−1^			425 × 10^3^ (338 × 10^3^–636 × 10^3^)
Mouse, TFV-DP, dermal tissue	6	100	
fmol mg^−1^			1,419 (750.1–2,561)
Sheep, TAF, plasma^d^	8	100	0.28 (0.21–0.47)
ng mL^−1^		
Sheep, TFV, plasma^d^	5	63	1.48 (1.28–1.96)
ng mL^−1^			
Sheep, TFV, dermal tissue	3	75	
ng mg^−1^			7.21	
fmol mg^−1^			425 × 10^3^		
Sheep, TFV-DP, dermal tissue	3	75	
fmol mg^−1^			706.2

TFV, tenofovir; TAF, tenofovir alafenamide.

a All values correspond to time points with the implant in place.

b Proportion of samples that contained quantifiable drug concentrations.

c Interquartile range, between first (25th percentile) and third (75th percentile) quartiles.

d Study days 7–14.

## Discussion

The availability of suitable animal models is of paramount importance in the preclinical development of biomedical drug delivery devices. Previous studies have reported the use of dogs (Gunawardana et al., 2015), rabbits (Su et al., 2019), and nonhuman primates (Chua et al., 2018; Su et al., 2019) in the evaluation of prototype TAF implants. Herein we report the use of mice and sheep for the first time, and discuss the advantages of these models.

The use of mice is desirable as a cost-effective model that can be extended to incorporate all major human hematopoietic lineages including T, B, monocyte/macrophage, dendritic, and natural killer cells. Humanized mouse (hu-mouse) models that are susceptible to vaginal and rectal HIV-1 infection can serve as a valuable complement to nonhuman primates in studying the PDs underlying HIV-1 PrEP. For example, we have used the bone marrow, liver, thymus (BLT) hu-mouse model to study the dose-response characteristics of the viral membrane-disrupting amphipathic peptide C5A in preventing vaginal and rectal HIV-1 infection (Gallay et al., 2018). We conducted a mechanistic study in BLT mice demonstrating empirically that TFV disoproxil fumarate and emtricitabine, the active agents in TRUVADA^®^, were slightly antagonistic in preventing HIV-1 acquisition vaginally and rectally (Gallay et al., 2017). These studies would not have been feasible in nonhuman primates because of the required group sizes. Here, we used C57BL/6J mice, as this species forms a fibrous overgrowth around implanted devices, mimicking the foreign body response observed in humans (Vegas et al., 2016).

The PK data shown in Figure 2 illustrate how the mouse model can be useful in studying systemic drug concentrations over time and local drug distribution for prototype subdermal implants delivering TAF. However, we were concerned that the high TAF dosing rate of 12 mg kg^−1^ d^−1^ (0.24 mg d^−1^ for 20 g mice) could lead to local drug saturation in the surrounding fluids, there-by limiting the control of drug release rate from the implant. If true, the mouse model would be of limited value in prototype device evaluation. Consequently, we evaluated TAF implants identical to those in the mouse studies in a large animal model of comparable body weight to humans. Sheep (32.3 ± 2.0 kg) were chosen for this study as they are significantly larger than beagle dogs (ca. 8–13 kg) and rhesus macaques (ca. 6–10 kg). In sheep, the implants delivered TAF at a similar rate as in mice. Had local drug saturation been a limiting factor in mice, much higher TAF release rates in sheep would have been expected.

This is the first reported use of sheep in the evaluation of a subdermal implant for HIV-1 PrEP. Sheep are docile, easy to handle, and have body mass (30 kg young, up to 80 kg adults) and anatomical similarities to humans (Sartoretto et al., 2016). Sheep studies have evaluated subcutaneous implants in isolated cases not related to HIV-1 PrEP (Halberstadt et al., 2002; Alexandre et al., 2014) and improved the understanding of toxicity related to injectable drug formulations (Asin et al., 2019). They also have been used for material biosafety testing and to study tissue foreign body response to implants (Rashid et al., 2004; Holt et al., 2013; Nezhad et al., 2016; Sartoretto et al., 2016). While pigs have been used as a standard model in transdermal PK studies (Stricker-Krongrad et al., 2016), a standard for subcutaneous drug testing has not been established. Pigs are not ideal for subcutaneous toxicity and PK studies because they have a thick subcutaneous fatty layer (Moyo et al., 2018) that can complicate implant evaluation–such as palpability (Kim et al., 2019) and visualization by ultrasound imaging (McEvoy et al., 2007)– and make drawing blood under restraint difficult. Additionally, they are uncooperative, and can grow from 25 to 100 kg within 8 weeks (Rashid et al., 2004). This rapid weight gain can complicate PK analysis.

The TAF implants appeared safe and well-tolerated in both species based on clinical observations and histologic evaluation of the implant pocket, although longer studies would be required to thoroughly evaluate safety. Noninvasive ultrasonic imaging of the implants *in vivo* (sheep) correctly measured the implant dimensions (length, 10.5 mm; dia., 2.34 mm). No accumulation of fluid around the implants was observed by ultrasound during the study. No signs of tissue irritation by ultrasound were noted, including no accumulation of fluid around the implants, no dermal thickening, and no sign of inflammatory infiltrates (diffuse hyper-echogenicity of the fatty layer/hypodermis).

Drug and drug metabolite concentrations were analyzed in plasma and dermal tissues adjacent to the implant. Future studies also will include the measurement of analyte concentrations in peripheral blood mononuclear cells (PBMCs) as well as vaginal, rectal, and lymphatic tissues. Median systemic TFV concentrations at equilibrium in sheep were 146 times lower than in mice, while allometric scaling predicts a factor of 253 (TAF release rate, 0.27 mg d^−1^, exponent, 0.75). The 1.7-fold difference between predicted and measured plasma TFV exposure in these vastly different animal models (in terms of body mass and metabolism) is encouraging.

While the implants delivered TAF at similar rates in mice and sheep, the local drug and drug metabolite concentrations in local dermal tissues were remarkably different (Table 1; Figure 2), suggesting that drug clearance is dependent on body weight and animal species. The median dermal tissue TFV concentrations were 17 times higher in mice, but the TFV-DP concentrations were only double in mice, suggesting a saturation of the kinases involved in mono- and diphosphorylation of TFV. The interspecies comparison also is supported by the median mole fraction of TFV-DP as a function of the total measured TFV concentrations (TFV + TFV−DP) by animal species: mice, 0.25%; sheep, 2.6%.

Drug and drug metabolite concentrations in tissues adjacent to the implant are the likely drivers of local tolerance and, hence, device safety. Because oral TAF is an FDA-approved regimen, the primary safety concern with a TAF implant will be local, not systemic toxicity. Understanding the distribution and clearance of TAF and its metabolites in the tissues proximal to the implant as a function of animal model therefore is important in the preclinical development phase. Prior to this study, little was known on the local accumulation of these compounds across species.

The findings presented have important implications when PK data from various animal models are extrapolated to PD effects, in terms of local toxicity and putative efficacy in HIV-1 prevention.

## Data Availability Statement

The raw data supporting the conclusions of this article will be made available by the authors, without undue reservation.

## Ethics Statement

The animal studies was reviewed and approved by The Scripps Research Institute, University of Texas Medical Branch at Galveston.

## Author Contributions

MG, MR-C, DS, SW, PG, RF, AC, JK, KV, and MM conducted experiments. MB, MG, JM, PAG, KV, and MM designed experiments, and MG, RF, AC, JK, KV, and MAM collected data. MB, PW, JM, DW, and CH interpreted data. MB drafted the manuscript, and JM and CH contributed to manuscript editing. All authors have given approval to the final version of the article.

## Funding

Research reported in this publication was supported by the National Institute of Allergy and Infectious Diseases of the National Institutes of Health under Award Number R01AI120748. The content is solely the responsibility of the authors and does not necessarily represent the official views of the National Institutes of Health. The sheep study was carried out using internal, discretionary funds that are gratefully acknowledged.

## Conflict of Interest

Author DW was employed by the company Approva Consulting LLC.

The remaining authors declare that the research was conducted in the absence of any commercial or financial relationships that could be construed as a potential conflict of interest.
